# The Problems Experienced by Employees with Chronic Disease during the COVID-19 Pandemic

**DOI:** 10.3390/ijerph19010578

**Published:** 2022-01-05

**Authors:** Dilaver Tengilimoğlu, Uğur Gönüllü, Oğuz Işık, Nurperihan Tosun, Aysu Zekioğlu, Onur Tengilimoğlu, Mustafa Younis

**Affiliations:** 1 Department of Management, Faculty of Management, Atılım University, Gölbaşı, Ankara 06830, Turkey; dilaver.tengilimoglu@atilim.edu.tr; 2 Department of Chest Disease, Faculty of Medicine, Atılım University, Gölbaşı, Ankara 06830, Turkey; ugur.gonullu@atilim.edu.tr; 3 Department of Health Management, Faculty of Economics and Administrative Sciences, Hacettepe University, Ankara 06100, Turkey; oguz.isik@gmail.com; 4 Department of Health Management, Faculty of Health Sciences, Cumhuriyet University, Sivas 58140, Turkey; nurperihankarabulut@gmail.com; 5 Department of Health Management, Faculty of Health Sciences, Trakya University, Edirne 22030, Turkey; 6 Department of Gynecology&Obstetrics, Faculty of Medicine, Istanbul University, Istanbul 34452, Turkey; onurtengilimoglu@gmail.com; 7 Department of Health Policy and Management, Jackson State University, Jackson, MS 39217, USA; younis99@gmail.com

**Keywords:** chronic diseases, COVID-19, employee, work life, Turkey

## Abstract

Chronic diseases served as a silent global epidemic before the pandemic, and individuals living with chronic disease now form one of the groups most affected by COVID-19. This study aims to determine the problems that employees with chronic disease face during the COVID-19 pandemic. As part of the study, data were collected from 952 individuals who live with chronic disease in Turkey. Of these, 76.6% of respondents worked for the public sector, a large majority of whom (67.7%) have worked full time during the COVID-19 pandemic. It was found that the COVID-19 fear level of employees living with chronic disease was higher than moderate (21.061 ± 7.607). When the variables affecting the COVID-19 fear level are listed in order of relative significance, eating problems, residing in the Mediterranean region, having asthma, and working as a female employee made the greatest impact, respectively. Necessary conditions of work should be provided to those living with chronic disease who could adapt themselves to working flexibly or working from home, so that they would not feel isolated from business life. This group should be provided with essential protective equipment, their working conditions must be reviewed and vaccination priority could be given to them.

## 1. Introduction

In the 21st century, the global burden and threat of chronic diseases are considered as a major public health problem that undermines sustainable social and economic development worldwide. Reducing the global disease burden has become an imperative for global development [1,2,3].

Though epidemiological data shows different rates of chronic diseases and multimorbidity among patients affected by COVID-19 in various countries and regions [4], it is stated [5,6] that patients with such chronic diseases as obesity, diabetes, hypertension, cardiovascular diseases and chronic kidney failure face a higher risk of severe complications and death due to COVID-19. Individuals with chronic disease have been affected by the COVID-19 pandemic both directly and indirectly [5]. The COVID-19 pandemic creates a direct and worrisome risk for individuals with chronic disease [7].

Chronic diseases are thought to have played a critical role in triggering more than 1 million deaths from COVID-19 to date [8]. In addition to morbidity and mortality in chronic diseases, the COVID-19 pandemic has created social isolation, disruption of lives within the scope of stay-at-home measures, and social and economic difficulties [9]. In managing chronic diseases, regular monitoring, treatment follow-up, control, and reduction of risk factors associated with these diseases are significant [10]. During the pandemic, chronic patients’ concerns about safe access to healthcare have increased while their ability to prevent or control chronic diseases has decreased [11]. Furthermore, health institutions have redirected their capacities to pandemic, acute and emergency health services [12].

Due to the risk of infection transmission and the need to reallocate resources to deal with the growing number of COVID-19 patients, elective surgeries, outpatient appointments, and cardiac imaging have mostly been canceled worldwide [13]. Because of fear of the life-threatening COVID-19 pandemic, namely the fear of infection, or the restrictive policies of governments, outpatients’ hospital visits decreased significantly, and patients missed their appointments [14] or did not come for their routine check-ups [15,16]. Inability to effectively manage chronic diseases during the pandemic process, interruption of routine care, delays in procedural treatments, increased stress levels, interruption of diet, daily activity, and physical activity [17] may result in delays in diagnosis and treatment [18], and health consequences such as decline in life quality and increased mortality [19]. Health systems have benefited from telemedicine technology to monitor chronic diseases, but telemedicine is used more frequently in high-income countries as it is highly dependent on the availability of technology and expertise [20]. Measures such as curfews and quarantines during the COVID-19 pandemic cause serious negative impacts on countries’ economies. These measures lead to the fact that cancer and chronic patients themselves or their family members experience psychosocial stress resulting from job or income loss and have difficulties in accessing inpatient services [21,22].

Medical services have been disrupted in part or as a whole in many countries during the pandemic. In a study conducted in Belgium, it was found that the health system capacity was being shifted to services to treat COVID-19, while chronic care services decreased and fewer consultation services were being provided [23]. In addition, it is known that some patients face socio-economic problems, lose their health insurance, and have difficulties in hospital applications and access to medication, particularly due to the restrictions imposed during the pandemic. It is noted that the online or face-to-face visits made to chronic patients have halved in the USA, and similarly, chronic patients face challenges in accessing medical services in Italy and India due to the lockdowns imposed [7,24,25].

In order to eliminate the risks to employees who have chronic disease during the pandemic, several precautions have been taken in Turkey. Flexible remote working and flexible working hours have been brought to both public and private sectors in Turkey as part of the struggle with the pandemic. Under the Circular released by the Turkish Presidency numbered 2020/8, it was resolved that those working for public institutions and organizations (except for the Health Ministry and the National Intelligence Organization), aged 60 and over, with chronic disease as set out by the Health Ministry, would be deemed on administrative leave. In the struggle with the pandemic, the re-allocation of the resources reserved for medical services may negatively affect the care of patients not infected with the coronavirus. Measures such as social distancing and other restrictions, intended for mitigating the spread of COVID-19, may affect the standard care provided to individuals with chronic disease [26]. This study aims to determine the problems experienced by individuals with chronic disease during the COVID-19 pandemic and to identify the factors affecting their COVID-19 fear levels.

## 2. Literature Review

Chronic diseases, which are the cause of 71% of all deaths worldwide [27], have devastating health consequences for individuals, families, and societies, as well as creating serious financial and economic risks to the economies of developed and developing countries and threatening the sustainability of health systems [28]. It has been found that more than 60% of some chronic patient populations, such as those with cancer, cardiovascular disease, and stroke, have catastrophic health expenditures [29]. Chronic diseases account for approximately 80% of all years of disability around the world [30]. Of the chronic diseases responsible for 77% of deaths in low-income and middle-income countries, the ones that cause the most death and disability globally are cardiovascular diseases (such as heart attack and stroke), cancer, chronic respiratory diseases (such as chronic obstructive pulmonary disease and asthma), and diabetes [27]. Harvard School of Public Health (HSPH) predicts that the economic burden of life lost due to all chronic diseases will be USD $43.3 trillion in 2030. Between 2011 and 2013, the lost output from cancer, cardiovascular disease, chronic respiratory diseases, diabetes, and mental illnesses was estimated to be approximately USD $47 trillion, which represented 75% of global GDP in 2010 [31]. In the USA, the economic burden of all chronic diseases is expected to be USD 265,000 per capita between 2015 and 2050 [32].

Chronic diseases put a strain on countries’ social welfare and health care systems. They can cause reduced workplace productivity, long-term disability, dwindling family finances, and, in the long run, a considerable decline in countries’ productive capacity [33]. In the EU, approximately 23.5% of the working population is known to have a chronic disease. Europe has the highest burden of chronic disease, which accounts for 86% of all deaths and is a significant cause of morbidity and disability estimated in disability-adjusted life years [34]. Encouraging the well-being, health, and job participation of employees with chronic diseases will improve their employability, well-being, health level, and productivity while lowering absenteeism [35], personnel turnover, health care expenditures, and occupational health care costs [36].

This study provides important data revealing the problems and expectations of individuals with chronic diseases, one of the groups disadvantaged by working under pandemic conditions.

## 3. Methods

The population of this study is comprised of people actively working in a job and having at least one chronic disease. It was found that a minimum of 664 people should be included in the research for a confidence level of 99% and the error margin stands at ±0.05. A total of 952 people gave a response as part of this study. A pilot study was not conducted. Data were collected through a convenience sampling method.

A survey was used as the data collection tool for this study. Since it was not practicable to conduct the survey face-to-face during the COVID-19 pandemic, the survey used in this study was prepared with the help of RedCap (Research Electronic Data Capture), a web application and a platform to collect, manage and share data. Besides including statements that identify the socio-demographic characteristics of employees with chronic disease, the e-survey encompassed statements about the participants’ chronic diseases and the problems they have experienced socially and in relation to medical services. The last section of this e-survey involved the fear of COVID-19 scale developed by Ahorsu et al. (2020) [37] to measure the COVID-19 fear levels of people with chronic disease. The scale is a unidimensional 7-item structure. The participants indicate their level of agreement with the statements using a five-item Likert-type scale. Answers included “strongly disagree,” “disagree,” “neither agree nor disagree,” “agree,” and “strongly agree”. The minimum score possible for each question is 1, and the maximum is 5. A total score is calculated by adding up each item score (giving a range from 7 to 35). The higher the score, the greater the fear of COVID-19. While assessing the findings, it should not be forgotten that the research data was collected online.

The data was collected in December 2020. Before initiating the research, ethical permission was granted by the Human Research Ethics Committee of the Atılım University.

The SPSS (23) statistics (BMI, Armonk, NY, USA) program was used for data analysis. Before proceeding with the analysis, the validity and reliability of the Fear of COVID-19 Scale were evaluated through confirmatory factor analysis (CFA) and Cronbach alpha coefficient, respectively. It was found as a result of CFA that the cohesion criteria regarding the unidimensional 7-item model (CMIN = 40.113 Df = 9; *p* = 0.000; CMIN/DF = 4.457; RMR = 0.038; GFI = 0.989; NFI = 0.990; TLI = 0.982; CFI = 0.992; RMSEA = 0.060) indicated good cohesion, and the factorial coefficients of the statements fell into the interval 0.64–0.83. The Cronbach alpha coefficient was found to be 0.901.

Besides descriptive statistics like frequency, percentage, average and standard deviation, linear regression analysis was used to identify the factors affecting the COVID-19 fear levels of individuals with chronic disease.

## 4. Results

Table 1 shows the socio-demographic characteristics of the research participants. Females constituted 59.8% of all participants. Most participants (77.6%) were over 40 years old, and 85.3% had completed undergraduate and graduate programs of universities. According to the distribution of respondents by region, the highest participation in this research came from those living in the Central Anatolia Region (30.8%), Marmara Region (20.9%), and Aegean Region. Of all participants, 76.6% worked for the public sector, most of whom (67.7%) worked full time during the pandemic. 49.8%of overall participants said that the measures taken by the state were inadequate. 35.7% stated that these measures were relatively adequate, while 14.5% found the measures adequate. In general, the COVID-19 fear levels of employees with chronic disease were found to be moderate (21.061 ± 7.607) (Table 1).

Table 2 shows information with regard to the medical conditions of the participants. A total of 17% of the participants were diagnosed with COVID-19. Of all participants, 35.7% were diagnosed with hypertension, 23.4% with diabetes, 16.6% with thyroid disorder, 16.1% with cardiovascular diseases, 14.8% with asthma, 14.1% with COPD, and 6.8% with arthritis. Furthermore, it was reported that 62% of the overall participants had at least one of these diseases, while 24.2% had at least two of them, and 3.9% were diagnosed with three or more of these diseases. 32.2% of the participants stated that they faced medical issues other than the chronic diseases they had already been diagnosed with during the COVID-19 pandemic. 28.8% of participants reported that they postponed seeking medical services during the pandemic. The mass body index of the participants was measured, and it was found that most of them were overweight (45%) and obese (24.4) (Table 2).

Table 3 demonstrates the problems faced by the participants in their social lives and in the medical services offered during the COVID-19 pandemic. The major social life problem was the inability to see family members and relatives (59%), inability to meet with social circles (56.6%), and inability to undertake or postponement ofsports/training (54.1%). Moreover, 15.7% of the participants stated that they had a loss of income. The major problem seen in medical services was the inability to go for a regular check-up regarding respondents’ chronic diseases (54.8%). 44.1% noted that they could not have their regular check-ups and examinations, due to the pandemic. In addition, 36% of the overall participants stated that they had to postpone receiving the medical services they had needed due to the pandemic, while 17.2% and 3.7% could not obtain medical services and/or had problems accessing the medication they had already been using, respectively (Table 3).

Table 4 includes the multiple linear regression results identifying the employees’ COVID-19 fear levels as affected by their socio-demographic characteristics and health conditions, as well as the challenges they faced in social and medical services. The Durbin–Watson coefficients for the regression model are less than 2.5 and the variation inflation factor (VIF) is less than 10, pointing to the fact that there is no multiple correlation and autocorrelation (Hair et al., 2010). The statistical estimations related to the regression model have shown the model to be significant (*p* < 0.001). The variables used in the research have shown that the total variance in the COVID-19 fear levels among the employees is 17.1%.

The results showed that the socio-demographic characteristics of gender (t_female_ = 3.028; *p* = 0.003), education level (t_graduate_ = −3.084; *p* = 0.002), and the region of residence (t_mediterranean_ = 3.290; *p* = 0.001) of employees affected their COVID-19 fear levels. The fear levels of female employees were higher than those of male employees. Those living in the Mediterranean region showed a higher fear level than those residing in the Central Anatolia region. On the other hand, graduate employees indicated lower COVID-19 fear levels than undergraduate ones. As for the impact of employees’ chronic diseases on COVID-19 fear levels, it was found that employees with asthma (t = 2.435; *p* = 0.015) demonstrated higher COVID-19 fear levels than those without asthma. The COVID-19 fear levels of employees who struggled with a disease other than the current chronic one (t = 2.258; *p* = 0.024) were higher than those who did not. When we examined the impact of the problems arising from the pandemic on the COVID-19 fear levels, we found that employees having eating problems such as overeating or lack of appetite (t = 4.038; *p* < 0.001) indicated higher COVID-19 fear levels than those who did not have such problems. When it comes to the social problems resulting from the pandemic, it was seen that employees who were unable to see their family members or relatives (t = 2.192; *p* = 0.029) indicated higher COVID-19 fear levels than those who were able to. Employees who faced a loss of income (t = 3.081; *p* = 0.002) due to the pandemic showed higher COVID-19 fear levels than those who did not. When the variables affecting the COVID-19 fear level are listed in order of relative significance, it can be said that eating problems like overeating or lack of appetite (β = 0.135), residing in the Mediterranean region (β = 0.114), having asthma (β = 0.112), and working as a female employee (β = 0.106) made the greatest impact (Table 4).

## 5. Discussion

The COVID-19 pandemic, persisting across the world for more than a year, has badly affected many areas of life, ranging from economic structures to the social order within countries. Restrictions have been brought in to prevent the destructive impacts of the pandemic and to take control of the process. New working methods and alternative service delivery models have been developed. These arrangements have triggered a sweeping change ranging from social relations to business lives. This is why this study investigated the problems experienced by employees with chronic disease during the pandemic.

As part of the study, 64.7% of respondents said that they worked full time during the COVID-19 pandemic. In contrast, 15.7% of overall respondents stated that they faced a loss of income. A study carried out with the participation of 369 adults living in 65 different Chinese provinces highlighted that 28% of participants lost their jobs and 38% started to work from home. This new working arrangement was found to adversely affect the mental health of employees [38]. A study of working mothers in Italy found out that 66.5% of them abandoned their jobs or started working from home [39]. It was soon understood that different countries adopted different work arrangements during the pandemic. However, it was seen that working from home was heavily preferred. On the other hand, it was also reported that respondents faced a loss of income. In this study, most of the participants reported that they had been working for the public sector, a situation enabling the continuation of full-time work.

When the variables affecting their COVID-19 fear level are listed in order of relative significance, it can be said that eating problems like overeating or lack of appetite, having asthma, and working as a female employee made the greatest impact, respectively. It was found that eating disorders had an impact on COVID-19 fear levels. Although there are different research findings on the relationship between COVID-19 and asthma in the literature [40], there are also studies noting that there exists a relationship between severe asthma phenotypes and the adverse clinical results of COVID-19 [41,42]. It was understood in this research that residing in the Mediterranean region had an impact on COVID-19 fear levels. With the normalization process after May 2020, 3,256,568 tourists [43] visited the Mediterranean region. This may have contributed to an increased COVID-19 fear level in the region. Furthermore, it was found that there was a statistically significant correlation between residing in the Mediterranean region and developing hypertension, asthma, and arthritis (*p* < 0.05).

Although studies indicated that male gender is a risk factor for COVID-19 [38,44], the female employees participating in this study were found to have higher COVID-19 fear levels. This can be explained by the fact that besides their role in business life, there also exist other pressures on many women resulting from the responsibilities of being a spouse and a mother. Moreover, since the life expectancy of women is longer than that of men, women are more likely to have lost their husbands and live alone. With less social assistance, this may increase fear of COVID-19 infection among women.

When the COVID-19 fear level was brought into focus, it was found that the participating employees with chronic disease showed a moderate level of fear of COVID-19. A study undertaken with the participation of 263 individuals in China suggested that 52.1% of the participants were deeply concerned or terrified due to the COVID-19 pandemic. On the other hand, 69.2% of the participants stated that work stress had increased [45]. The study results revealed that fear was triggered and work-related stress was increased during the pandemic.

In this study, it was noted that the participating employees with chronic disease had had no difficulty in accessing medication during the pandemic. A baseline study was carried out in China focusing on community pharmacies, and it was stated that those with chronic disease were the most vulnerable group during the pandemic when it comes to access to medications. Similar to one result of this research, it was reported in the baseline study conducted in China that access to medication had not been hampered by the use of community pharmacies [46]. It may be suggested that measures taken for individuals with chronic disease whose access to medication had gained more importance due to the pandemic addressed problems adequately.

In the 21st century, the COVID-19 pandemic has emerged as an important public health crisis and has had economic, social, and many other effects. In light of the information revealed in this descriptive study, making arrangements regarding employment and working conditions for disadvantaged employees in future pandemics will enable decision-makers and politicians to make healthier decisions.

## 6. Conclusions

As the struggle with the COVID-19 pandemic continues, the impacts of this pandemic on social and business life come to the surface. The shift from working in the workplace to working from home and, depending on the changing order, the conflicts between business and social roles are the biggest impacts on an individual’s life. It is beyond any doubt that one group most affected by the pandemic is comprised of employees with chronic disease who try hard to cling to their jobs to escape the destructive economic side of the pandemic and avoid loss of income. All countries have started to take measures to bar this specific group from getting infected and to provide these individuals with continuous access to services such as diagnosis, treatment and medication. In this framework, providing administrative leave for employees with chronic diseases such as cancer that affect the immune system is a significant step forward. However, necessary conditions of work should be provided to those living with chronic disease who could adapt themselves to working flexibly or working from home so that they would not feel isolated from business life.

Employees with chronic illnesses who continue to work at home can access healthcare professionals without going to the hospital, by the help of telemedicine or mobile health applications. Online appointment payments can be covered under social security. In this way, while individuals are protected against the risk of COVID-19, the work of employees with a chronic disease will not be disrupted.

Employees with chronic diseases should be given priority during vaccination programs to keep up the pace of production and to protect the health of these individuals, regardless of which sector they work in. During this process, psycho-socio assistance should be given to these individuals who fear losing their job, while trying to manage chronic disease and to handle the stress that stems from fear of infection. In this context, it is important to expand the use of such applications as telemedicine and home care services. It is also proposed that similar policies should be followed for employees with chronic disease without making any distinction between the public and private sectors. Last but not least, this group should be provided with essential protective equipment, their working conditions must be reviewed and vaccination priority could be provided for them.

The limitation of the present study is that it was carried out with a structured scale. In future studies, it will be important to examine the suggestions and expectations of employees with chronic diseases in terms of policies to be put forward.

## Figures and Tables

**Table 1:** The Socio-Demographic Characteristics of Participants.

				*n*	%
Gender	Male			383	40.2
	Female			569	59.8
Age (46.44 ± 9.29)	≤30			74	7.8
	31–40			140	14.7
	41–50			435	45.7
	51–60			252	26.5
	≥61			51	5.4
Education	Primary			18	1.9
	Secondary			122	12.8
	Undergraduate			605	63.6
	Graduate			207	21.7
Region	Mediterranean			114	12.0
	Eastern Anatolia			64	6.7
	Aegean			118	12.4
	Southeastern Anatolia			48	5.0
	Central Anatolia			293	30.8
	Black Sea			116	12.2
	Marmara			199	20.9
Sector	Public			729	76.6
	Private			223	23.4
Working conditions during pandemic	Full Time			615	64.7
	Flexibly			202	21.3
	Home-Online			91	9.6
	Not specified			42	4.4
Opinion on the measures taken by the state during pandemic	Inadequate			474	49.8
	Partially			340	35.7
	Adequate			138	14.5
	Min	Max	Mean	S.D	
COVID-19 fear levels	5	35	21.061	7.607	

**Table 2:** Medical conditions of participants.

		*n*	%
Diagnosed with COVID-19	No	790	83.0
	Yes	162	17.0
Chronic disease	Hypertension	340	35.7
	Diabetics	223	23.4
	Thyroid	158	16.6
	Cardiovascular disease	153	16.1
	Asthma	141	14.8
	COPD	134	14.1
	Arthritis	65	6.8
Number of chronic diseases	1 disease	590	62.0
	2 diseases	230	24.2
	3 and more diseases	132	13.9
How often did you get a check-up for your chronic disease?	Monthly	41	4.3
	3 months	209	22.0
	6 months	247	25.9
	Yearly	307	32.2
	I don’t go periodically	148	15.5
Did you experience any health problems other than your chronic disease during the pandemic?	No	645	67.8
	Yes	307	32.2
Which health institution did you go to for both your chronic illness and other health problems during the pandemic?	Postponed	274	28.8
	Public hospital	242	25.4
	Private hospital	172	18.1
	Family doctor	115	12.1
	University hospital	113	11.9
	Others (medical clinic, etc.)	36	3.8
BMIw	Underweight	16	1.7
	Normal	276	29.0
	Overweight	428	45.0
	Obese I	201	21.1
	Obese II	31	3.3

**Table 3:** Problems caused by the pandemic.

		*n*	%
Medical services	Inability to go to check-up for chronic disease (HP1)	545	57.2
	Nutrition problems (overeating or lack of appetite) (HP2)	522	54.8
	Inability to have examinations and tests for chronic disease (HP3)	420	44.1
	Postponements in health services (HP4)	343	36.0
	I could not meet my healthcare needs (HP5)	164	17.2
	Access problem for drugs (HP6)	35	3.7
Social life	I could not see my family and relatives (SocialP1)	562	59.0
	I could not meet with my social circle (SocialP2)	539	56.6
	Inability to access/postponement of sports/training (SocialP3)	515	54.1
	Loss of income (SocialP4)	149	15.7

**Table 4:** The multiple linear regression results identifying the employees’ COVID-19 fear levels as affected by their socio-demographic characteristics and health conditions.

	Predictors	B	S.E.	β	t	*p*	VIF
	(Constant)	12.286	2.057		5.971	0.000	
Socio-demographic characteristics	Male (Reference)						
	Female	1.651	0.545	0.106	3.028	0.003	1.360
	Age	0.049	0.028	0.060	1.729	0.084	1.311
	Undergraduate (Reference)						
	Primary	2.445	1.803	0.044	1.356	0.175	1.154
	Secondary	0.085	0.739	0.004	0.116	0.908	1.157
	Graduate	−1.902	0.617	−0.103	−3.084	0.002	1.233
	Central Anatolia (Reference)						
	Mediterranean	2.654	0.807	0.114	3.290	0.001	1.311
	Eastern Anatolia	1.704	0.998	0.056	1.707	0.088	1.195
	Aegean	0.955	0.793	0.041	1.204	229	1.307
	Southeastern Anatolia	1.404	1.149	0.040	1.222	0.222	1.185
	Black Sea	1.414	0.805	0.061	1.756	0.079	1.316
	Marmara	0.923	0.663	0.049	1.393	0.164	1.387
	Sector of Work	1.166	0.636	0.065	1.834	.067	1.380
	Full time (Reference)						
	Flexibly	0.209	0.609	0.011	0.343	0.732	1.186
	Home-Online	−0.858	0.850	−0.033	−1.009	0.313	1.194
	Not specified	0.657	1.161	0.018	0.566	0.572	1.087
Health Data	BMI	0.011	0.062	0.006	0.170	0.865	1.273
	Diabetes	0.240	0.668	0.013	0.360	0.719	1.527
	Hypertension	0.713	0.618	0.045	1.154	0.249	1.668
	Cardiovascular	0.922	0.731	0.044	1.261	0.208	1.370
	Asthma	2.396	0.984	0.112	2.435	0.015	2.336
	Thyroid	1.035	0.749	0.051	1.380	0.168	1.486
	Arthritis	0.458	1.016	0.015	0.451	0.652	1.238
	1 Disease (Reference)						
	2 Diseases	0.569	0.704	0.032	0.808	0.419	1.733
	3 and more diseases	−1.161	1.143	−0.053	−1.016	0.310	2.980
	Other Health Problem	1.193	0.529	0.073	2.258	0.024	1.161
Problems Caused by the Pandemic	HP1_Checkup	−1.090	0.607	−0.071	−1.797	0.073	1.715
	HP2_Nutritional	2.061	0.510	0.135	4.038	0.000	1.229
	HP3_Dissection	0.911	0.622	0.059	1.464	0.144	1.819
	HP4_Delay	0.047	0.528	0.003	0.089	0.929	1.226
	HP5_NeedforHC	0.962	0.694	0.048	1.386	0.166	1.312
	HP6_MedicamentAccess	0.278	1.278	0.007	0.218	0.828	1.106
	Social_P1	1.238	0.565	0.080	2.192	0.029	1.469
	Social_P2	0.399	0.560	0.026	0.713	0.476	1.469
	Social_P3	−0.726	0.487	−0.048	−1.489	0.137	1.124
	Social_P4	2.095	0.680	0.100	3.081	0.002	1.166

R = 0.414; R^2^: 0.171; F = 5.391; *p* < 0.001; Durbin-Watson = 1.967.

## Data Availability

Selected data may be made available upon reasonable request.

## References

[1] Kankeu H.T., Saksena P., Xu K., Evans D.B. (2013). The financial burden from non-communicable diseases in low-and middle-income countries: A literature review. Health Res. Policy Syst..

[2] WHO (2013). Sixty-Sixth World Health Assembly: Follow-Up to the Political Declaration of the High-level Meeting of the General Assembly on the Prevention and Control of Non-communicable Diseases.

[3] Nugent R., Bertram M.Y., Jan S., Niessen L.W., Sassi F., Jamison D.T., Pier E.G., Beaglehole R. (2018). Investing in non-communicable disease prevention and management to advance the Sustainable Development Goals. Lancet.

[4] Martini N., Piccinni C., Pedrini A., Maggioni A. (2020). COVID-19 and chronic diseases: Current knowledge, future steps and the MaCroScopio project. Recenti Progress. Med..

[5] Liu N., Huang R., Baldacchino T. (2020). Telehealth for non-critical patients with chronic disease during the pandemic. J. Med. Internet Res..

[6] Wang B., Li R., Lu Z., Huang Y. (2020). Does comorbidity increase the risk of patients with COVID-19: Evidence from meta-analysis. Aging.

[7] Hartmann-Boyce J., Morris E., Goyder C., Kinton J., Perring J., Nunan D., Mahtani K., Buse J.B., del Prato S., Ji L. (2020). Diabetes and COVID-19: Risks, Management, and Learnings From Other National Disasters. Diabetes Care.

[8] IHME (2020). GBD Results Tool. http://ghdx.healthdata.org/gbd-results-too.

[9] Hacker K.A., Briss P.A., Richardson L., Wright J., Petersen R. (2021). COVID-19 and Chronic Disease: The Impact Now and in the Future. Prev. Chronic Dis..

[10] Chen X., Zhou X., Li H., Li J., Jiang H. (2020). The value of WeChat application in chronic diseases management in China. Comput. Methods Programs Biomed..

[11] Czeisler M.É., Marynak K., Clarke K.E.N., Salah Z., Shakya I., Thierry J.M., Ali N., McMillan H., Wiley J.F., Weaver M.D. (2020). Delay or avoidance of medical care because of COVID-19–related concerns—United States, June 2020. MMWR Morb. Mortal. Wkly Rep..

[12] Barasa E.W., Ouma P.O., Okiro E.A. (2020). Assessing the hospital surge capacity of the Kenyan health system in the face of the COVID-19 pandemic. PLoS ONE.

[13] Adam S., Zahra S.A., Chor C.Y.T., Khare Y., Harky A. (2020). COVID-19 pandemic and its impact on service provision: A cardiology prospect. Acta Cardiol..

[14] Spalletta G., Porcari D.E., Banaj N., Ciullo V., Palmer K. (2020). Effects of COVID-19 Infection Control Measures on Appointment Cancelation in an Italian Outpatient Memory Clinic. Front. Psychiatry.

[15] Curigliano G., Cardoso M.J., Poortmans P., Gentilini O., Pravettoni G., Mazzocco K., Houss N., Pagani O., Senkus E., Cardoso F. (2020). Recommendations for triage, prioritization and treatment of breast cancer patients during the COVID-19 pandemic. Breast.

[16] Thirupathieswaran R., Prakash C.S., Krishnan R.S., Narayanan K.L., Kumar M.A., Robinson Y.H. (2021). Zero Queue Maintenance System using Smart Medi Care Application for COVID-19 Pandemic Situation. Proceedings of the 2021 Third International Conference on Intelligent Communication Technologies and Virtual Mobile Networks (ICICV) 2021.

[17] Saqib M.A.N., Siddiqui S., Qasim M., Jamil M.A., Rafique I., Awan U.A., Ahmad H., Afzal M.S. (2020). Effect of COVID-19 lockdown on patients with chronic diseases. Diabetes Metab. Syndr. Clin. Res. Rev..

[18] Gre Fegert J.M., Vitiello B., Plener P.L., Clemens V. (2020). Challenges and burden of the Coronavirus 2019 (COVID-19) pandemic for child and adolescent mental health: A narrative review to highlight clinical and research needs in the acute phase and the long return to normality. Child Adolesc. Psychiatry Ment. Health.

[19] Katsanos A.H., de Sa Boasquevisque D., Al-Qarni M.A., Shawawrah M., McNicoll-Whiteman R., Gould L., van Adel B., Sahlas D.J., Ng K.K.H., Perera K. (2021). In-hospital delays for acute stroke treatment delivery during the COVID-19 pandemic. Can. J. Neurol. Sci..

[20] WHO (2020). The Impact of the COVID-19 Pandemic on Noncommunicable Disease Resources and Services: Results of a Rapid Assessment. https://www.who.int/publications/i/item/9789240010291.

[21] Singh K., Kondal D., Mohan S., Jaganathan S., Deepa M., Venkateshmurthy N.S., Jarhyan P., Anjana R.M., Narayan K.M.V., Mohan V. (2021). Health, psychosocial, and economic impacts of the COVID-19 pandemic on people with chronic conditions in India: A mixed methods study. BMC Public Health.

[22] Guo Y., Shen M., Zhang X., Xiao Y., Zhao S., Yin M., Bu W., Wang Y., Chen X., Su J. (2021). Unemployment and Health-Related Quality of Life in Melanoma Patients during the COVID-19 Pandemic. Front. Public Health.

[23] Danhieux K., Buffel V., Pairon A., Benkheil A., Remmen R., Wouters E., van Olmen J. (2020). The impact of COVID-19 on chronic care according to providers: A qualitative study among primary care practices in Belgium. BMC Fam. Pract..

[24] Consonni M., Telesca A., Grazzi L., Cazzato D., Lauria G. (2020). Life with chronic pain during COVID-19 lockdown: The case of patients with small fibre neuropathy and chronic migraine. Neurol. Sci..

[25] Pal R., Bhadada S.K. (2020). COVID-19 and diabetes mellitus: An unholy interaction of two pandemics. Diabetes Metab. Syndr..

[26] Liang Y., Chang C., Chen Y., Dong F., Zhang L., Sun Y. (2020). Symptoms, Management and Healthcare Utilization of COPD Patients during the COVID-19 Epidemic in Beijing. Int. J. Chron. Obstruct. Pulmon. Dis..

[27] WHO (2021). Noncommunicable Diseases. https://www.who.int/news-room/fact-sheets/detail/noncommunicable-diseases.

[28] Vandenberghe D., Albrecht J. (2020). The financial burden of non-communicable diseases in the European Union: A systematic review. Eur. J. Public Health.

[29] Jan S., Laba T.-L., Essue B.M., Gheorghe A., Muhunthan J., Engelgau M., Mahal A., Griffiths U., McIntyre D., Meng Q. (2018). Action to address the household economic burden of non-communicable diseases. Lancet.

[30] IHME (2020). The Lancet: Latest Global Disease Estimates Reveal Perfect Storm of Rising Chronic Diseases and Public Health Failures Fuelling COVID-19 Pandemic. https://www.healthdata.org/news-release/lancet-latest-global-disease-estimates-reveal-perfect-storm-rising-chronic-diseases-.

[31] Bloom D.E., Cafiero E.T., Jané-Llopis E., Abrahams-Gessel S., Bloom L.R., Fathima S., Feigl A.B., Gaziano T., Mowafi M., Pandya A. (2011). The Global Economic Burden of Noncommunicable Diseases.

[32] Chen S., Kuhn M., Prettner K., Bloom D.E. (2018). The macroeconomic burden of noncommunicable diseases in the United States: Estimates and projections. PLoS ONE.

[33] Silvaggi F., Eigenmann M., Scaratti C., Guastafierro E., Toppo C., Lindstrom J., Rantala E., Imaz-Iglesia I., Barnfield A., Maassen A. (2020). Employment and chronic diseases: Suggested actions for the implementation of inclusive policies for the participation of people with chronic diseases in the labour market. Int. J. Environ. Res. Public Health.

[34] European Chronic Disease Alliance (2017). Joint Statement on “Improving the Employment of People with Chronic Diseases in Europe”. https://ec.europa.eu/health/sites/health/files/policies/docs/2017_chronic_framingdoc_en.pdf.

[35] Mandiracioglu A., Bolukbas O., Demirel M., Gumeli F. (2015). Factors related to presenteeism among employees of the private sector. Int. J. Occup. Saf. Ergon..

[36] Chordis Plus (2021). Chronic Diseases and Employment. http://chrodis.eu/08-chronic-diseases-and-employment/.

[37] Ahorsu D.K., Lin C.-Y., Imani V., Saffari M., Griffiths M.D., Pakpour A.H. (2020). The Fear of COVID-19 Scale: Development and Initial Validation. Int. J. Ment. Health Addict..

[38] Zhang S.X., Wang Y., Rauch A., Wei F. (2020). Unprecedented disruption of lives and work: Health, distress and life satisfaction of working adults in China one month into the COVID-19 outbreak. Psychiatry Res..

[39] Di Giorgio E., Di Riso D., Mioni G., Cellini N. (2021). The interplay between mothers’ and children behavioral and psychological factors during COVID-19: An Italian study. Eur. Child Adolesc. Psychiatry.

[40] Sunjaya A.P., Allida S.M., Di Tanna G.L., Jenkins C. (2021). Asthma and risk of infection, hospitalisation, ICU admission and mortality from COVID-19: Systematic review and meta-analysis. J. Asthma.

[41] Heffler E., Detoraki C., Contoli M., Papi A., Paoletti G., Malipiero G., Brussino L., Crimi C., Morrone D., Padovani M. (2021). COVID-19 in Severe Asthma Network in Italy (SANI) patients: Clinical features, impact of comorbidities and treatments. Authorea.

[42] Kow C.S., Capstick T., Hasan S.S. (2021). Are severe asthma patients at higher risk of developing severe outcomes from COVID-19?. Allergy.

[43] Association of Turkish Travel Acencies (2020). Turkey Tourism Statistical Report on the Latest Data. https://www.tursab.org.tr/statistics.

[44] Wolff D., Nee S., Hickey N.S., Marschollek M. (2021). Risk factors for COVID-19 severity and fatality: A structured literature review. Infection.

[45] Zhang Y., Ma Z.F. (2020). Impact of the COVID-19 pandemic on mental health and quality of life among local residents in Liaoning Province, China: A cross-sectional study. Int. J. Environ. Res. Public Health.

[46] Zheng S.Q., Yang L., Zhou P.X., Li H.B., Liu F., Zhao R.S. (2021). Recommendations and guidance for providing pharmaceutical care services during COVID-19 pandemic: A China perspective. Res. Soc. Adm. Pharm..

